# The time course of semantic ambiguity in visual word recognition: behavioral and ERP evidence for the lexical-semantic effect

**DOI:** 10.3389/fpsyg.2024.1378125

**Published:** 2024-07-09

**Authors:** Joonwoo Kim, Sangyub Kim, Kichun Nam

**Affiliations:** ^1^Department of Psychology, Korea University, Seoul, Republic of Korea; ^2^Department of Psychology, Chonnam National University, Gwangju, Republic of Korea; ^3^School of Psychology, Korea University, Seoul, Republic of Korea

**Keywords:** semantic ambiguity, word frequency, homonym, lexical-semantic processing, visual word recognition, ERP, N400, P600

## Abstract

**Introduction:**

Homonyms are words with multiple, unrelated meanings that share a single form and pronunciation. These words provide valuable insights into how semantic representation is retrieved and selected independently of orthography and phonology. This study aims to investigate the temporal dynamics of lexical and semantic processing in the visual recognition of Korean words. Specifically, we examine how homonyms and unambiguous words are processed differently during a lexical decision task (LDT) with EEG recording, considering the effects of word frequency and the number of meanings (NOMs).

**Methods:**

Participants performed a lexical decision task where they were required to determine if a visually presented stimulus was a valid Korean word. We compared the behavioral responses and event-related potentials (ERPs) evoked by homonyms and unambiguous words, each possessing either high or low word frequency. Both subjective and objective NOMs were measured and manipulated, while controlling for the subjective balance of individual frequencies of meanings to control for confounding from the relatedness of meaning (ROM). For ERP analysis, a non-parametric cluster-based permutation test was employed in addition to the two time windows of interest (i.e., N400 and P600).

**Results:**

Behavioral results indicated a marginally significant interaction between word frequency and semantic ambiguity along with a main effect of word frequency, showing faster and more accurate responses for high-frequency words. An ambiguity advantage was observed only for low-frequency words, with no significant effect found for high-frequency words. ERP results revealed that lexical-semantic interactions were reflected in the modulations of the N400 and P600 components. High-frequency homonyms elicited an enhanced N400 amplitude, while low-frequency homonyms showed a reduced P600 amplitude.

**Discussion:**

The findings suggest that the activation of semantic information occurs simultaneously with lexical processing, rather than during post-lexical or decision-making processes. Furthermore, considering balanced homonyms were employed in this study, inhibitory competition may arise from the high-frequency individual meanings of high-frequency words. In contrast, in low-frequency words, a facilitative effect may arise from gains in global semantic activation or semantic feedback to the orthographic level.

## Introduction

Ambiguity resolution presents a significant challenge for all languages, holding particular importance in the context of language modeling and understanding the human mind. Accordingly, semantic ambiguity has been one of the critical issues in the research of psycholinguistics over five decades (for recent reviews, see Eddington and Tokowicz, 2015; Rodd, 2018), with a particular interest in ambiguous words that encompass polysemes (e.g., bank), homographs (e.g., lead), homophones (e.g., flower–flour), heteronyms (e.g., read), and homonyms (e.g., bat). Among these various categories in ambiguous words, homonyms, words that involve multiple but distinct meanings encapsulated with shared form and pronunciation, prompt further exploration into how semantic, orthographic, and phonological information are represented and presented in the human brain.

The present study focused on Korean homonymy, aiming to investigate whether semantic ambiguity yields facilitative or inhibitory effects during visual word recognition. Given that the Korean script is characterized by its transparent orthography (Pae et al., 2019) and abundant ambiguity at the lexical level with ambiguous words amounting to 30% of modern words and expressions enlisted in the Korean corpus (Kang, 2005), Korean homonyms present an opportune terrain for investigating the semantic processing, particularly in the absence of potentially confounding phonological cues. Here, we also sought to examine potential interactions in the lexical and semantic processes, using a factorial design with two pivotal factors: word frequency and semantic ambiguity, in a lexical decision task (hereafter, LDT) where participants were instructed to judge if the presented stimulus is an existing word or not. Furthermore, to explore the time course of these lexical-semantic processes, we measured event-related potentials (ERPs) in electroencephalogram (EEG) recordings.

Since the pioneering research by Rubenstein et al. (1970), there has been a wide consensus that semantic ambiguity facilitates visual word recognition, namely ambiguity advantage (i.e., faster recognition of ambiguous words compared to unambiguous words). Support for the ambiguity advantage came primarily from empirical evidence employing LDT (Borowsky and Masson, 1996; Hino and Lupker, 1996; Pexman and Lupker, 1999). With respect to the loci of advantage and mental representation of ambiguous words, however, divergent explanations have been suggested. Initial localist accounts (Rubenstein et al., 1970; Jastrzembski, 1981) argued that an ambiguous word has multiple lexical representations (i.e., units) for each meaning, providing more converging evidence for the decision of lexicality in LDT, thus leading to faster response times compared to the single detector for an unambiguous word. Conversely, subsequent accounts (Balota et al., 1991; Borowsky and Masson, 1996; Hino and Lupker, 1996) have challenged the concept of one-to-one mapping between meaning and lexical representation, suggesting instead that an ambiguous word might possess multiple semantic representations within a single lexical entry. According to these accounts, an ambiguous word would benefit from the ample activation of semantic information from multiple semantic representations, compared with the lesser amount of activation from a single semantic representation of an unambiguous word. In the context of the connectionist framework (Seidenberg and McClelland, 1989; Plaut and McClelland, 1993), processing advantage of an ambiguous word was explained as a result of either global activation gains within the semantic level (Borowsky and Masson, 1996) or feedback from the semantic to the orthographic level (Balota et al., 1991; Hino and Lupker, 1996).

In contrast, some researchers have reported contradicting results demonstrating inhibitory effects of semantic ambiguity, or ambiguity disadvantage (i.e., slower recognition of ambiguous words compared to unambiguous words) (e.g., Rodd et al., 2002; Beretta et al., 2005; Klepousniotou and Baum, 2007). Rodd et al. (2002) pointed out that the effects of polysemes with multiple senses (i.e., related meanings) may have confounded with those of homonyms with multiple unrelated meanings in the prior literature that reported ambiguity advantage. Indeed, conflicting results were found for two different types of ambiguous words in those studies, in which polysemes (e.g., “twist”) yielded ambiguity advantage, while ambiguity disadvantage was shown for homonyms (e.g., “bark”). These results were further explained by Rodd et al. (2004), who developed the Parallel Distributed Processing model (PDP) proposed by Kawamoto (1993), in which each word is not represented by a single lexical entry nor by multiple semantic representations; instead, it consists of a unique pattern of activation across units at separate but inter-connected levels of form (i.e., orthography and phonology) and meaning (i.e., semantics). Thus, the ambiguity disadvantage for homonyms could be attributed to competition across unrelated meanings during semantic access, whereas the ambiguity advantage for polysemes may benefit from highly inter-correlated patterns of activation across the semantic units. It is noteworthy, however, that a number of LDT studies have found equivalent facilitative effects for both types of ambiguous words compared to unambiguous words (Pexman et al., 2004; Experiments 1 and 4; Hino et al., 2006, 2010; Haro and Ferré, 2018), thus replicating initial findings that reported the ambiguity advantage effect (e.g., Rubenstein et al., 1970; Jastrzembski, 1981). Moreover, similar results have also been reported in non-Indo-European languages including Korean (Yu and Nam, 2009), Chinese (Lin and Ahrens, 2010), and Japanese (Hino et al., 2006, 2010).

Concerning the ambiguity disadvantage effect, an alternative explanation suggested that this effect arises from difficulties in the task-specific decision-making processes. The “decision-making” account was supported by the studies that used tasks requiring more complete semantic processing compared to LDT, such as semantic relatedness judgment (Piercey and Joordens, 2000; Pexman et al., 2004) and semantic categorization (Hino et al., 2002, 2006). For instance, in a series of experiments (Experiments 2–3, and 5), Pexman et al. (2004) found an ambiguity disadvantage effect in the semantic relatedness judgment task. Interestingly, the processing cost for ambiguous words was found only on related trials, regardless of whether the meaning of the related pair word was dominant (e.g., bat-baseball) or subordinate (e.g., bat-vampire), while unrelated trials (e.g., bat-water) yielded no significant effect. The authors reasoned that if the ambiguity disadvantage were truly due to the semantic activation process, its effects would be observed in both related and unrelated trials. Instead, the results suggested that this inhibitory effect might be derived from response conflicts during decision-making processes.

Similarly, Hino et al. (2006), in a semantic categorization task, observed processing cost for ambiguous words with less related meanings (e.g., crane in living/non-living category judgment) but only in broad categories (i.e., living-object and human-related) but not in narrow ones (i.e., animal or vegetable). Specifically, they attributed the effect to the task-specific decision category, suggesting that broad categories require the retrieval of a large number of semantic features, while narrow categories require fewer features. Taken together, the decision-making accounts assumed that the inhibitory effect of semantic ambiguity arises when task-relevant decisions, either in semantic relatedness or judgment task, are more difficult to make. In contrast, other researchers challenged the view that the qualitative difference in the configuration of the response system gives rise to a discrete semantic ambiguity effect depending on the task (Rodd et al., 2002, 2004; Armstrong and Plaut, 2008, 2011). Instead, they posited that those differences in the cooperative and competitive dynamics lie in varying amounts of semantic settling and processing time during the semantic access, depending on the types of ambiguous words, but not on the task or the response system (Armstrong and Plaut, 2016).

However, the observed discrepancies in the semantic ambiguity effect across different studies may also be influenced by other factors. One possible explanation for these discrepancies is the relative frequency of each meaning within an ambiguous word (i.e., the degree to which it is balanced), particularly in the case of a homonym with multiple unrelated meanings. Balanced ambiguous words have equiprobable meanings (e.g., match-fire, match-game), while in unbalanced ones (e.g., toast-bread, toast-speech), a particular meaning among multiple meanings is used predominantly. Ambiguity studies, especially seeking how specific conditions influence the activation of meanings in ambiguous meanings, have manipulated the relative meaning frequency, using tasks involving priming and context (Klepousniotou and Baum, 2007; Bilenko et al., 2008; Klepousniotou et al., 2012; MacGregor et al., 2015; Brocher et al., 2018). Meanwhile, some studies have controlled for relative meaning frequencies by using either balanced or unbalanced ambiguous word stimuli or by comparing these two conditions as experimental variables (Mirman et al., 2010; Armstrong and Plaut, 2016). Researchers have observed different ambiguity effects for balanced and unbalanced words, with most agreeing that balanced homonyms incur a more pronounced processing cost compared to unbalanced homonyms (Rayner and Duffy, 1986; Duffy et al., 1988; Kawamoto, 1993; Kim and Nam, 2023), although a few have reported no significant difference between the two types (Forster and Bednall, 1976; Klepousniotou and Baum, 2007; Bilenko et al., 2008). Taken together, these findings demonstrated that, depending on the different types as well as the relative frequencies of meanings, ambiguous words might involve a distinct processing mechanism during recognition.

While ambiguity studies have often manipulated the relative meaning frequency, the word frequency, as one of the most robust factors in psycholinguistics, known as the word frequency effect, characterized by superior processing efficiency in high-frequency words (for a review, see Brysbaert et al., 2018), has typically been controlled for across conditions to eliminate its potential confounding effects on the variables of interest. Consequently, word frequency is less used as an experimental variable, although word frequency has been observed to interact with various factors, resulting in different patterns for high and low-frequency words. To the best of our knowledge, two studies conducted LDT experiments where the effect of semantic ambiguity was examined while manipulating the word frequency of each word (Pexman et al., 2004; Kim et al., 2021). Both studies reported an interaction between word frequency and ambiguity, with an advantage effect found only for low-frequency homonyms. Meanwhile, a null effect (Pexman et al., 2004) and a marginally significant disadvantage effect (Kim et al., 2021) were reported for high-frequency homonyms. The explanation of these lexical-semantic interactions suggested that low-frequency homonyms may benefit from semantic feedback to orthographic activation, whereas in high-frequency words, responses are made even before the semantic activation, allegedly due to the word frequency effect (Pexman et al., 2004). Indeed, the word frequency effect has been suggested to occur rapidly even before semantic activation (Sereno and Rayner, 2003; Hauk et al., 2006).

In this regard, we manipulated the word frequency of both unambiguous and ambiguous words in an LDT with a factorial design, to explore the lexical-semantic interaction indicated in the two studies (Pexman et al., 2004; Kim et al., 2021). Furthermore, we employed the event-related potential (ERP) method, which allow us to explore the time course of lexical and semantic processes with its superior temporal resolution, providing a more detailed picture of how word frequency modulates the cognitive mechanisms underlying semantic processing. Specifically, we aimed to uncover whether the processing differences related to the semantic ambiguity occur in the lexical access stage, indexed by the word frequency effect, or independently during later stages.

Overall, although the weight of behavioral evidence has shed light on the semantic ambiguity effect, including its varying processing time for different types of ambiguous words (balanced/biased, polysemous/homonymous words), it remains vague regarding the specific processing stage at which these intricate semantic activation processes occur. Therefore, it calls for a more direct examination of the temporal loci of lexical access and semantic activation, employing methods with superior temporal resolution such as EEG (electroencephalogram) and MEG (magnetoencephalogram). Recent studies on semantic ambiguity have measured event-related potentials (ERPs) during EEG recording, with particular focus on the N400 component, a negativity that peaks around 400 ms after the stimulus presentation, known to reflect lexical-semantic effect modulated by variables such as word frequency and semantic ambiguity during word recognition (for a review, see Kutas and Federmeier, 2011).

A number of studies on semantic ambiguity have examined the N400 effect for homonyms, using the priming paradigm (Atchley and Kwasny, 2003; Swaab et al., 2003; Klepousniotou et al., 2012; MacGregor et al., 2015), wherein reduced N400 amplitude compared to the unrelated condition (i.e., N400 priming effect) was examined for each condition. For instance, Klepousniotou et al. (2012) investigated whether a discrete pattern of N400 priming effect was elicited by various types of ambiguous words including balanced or unbalanced homonyms and metaphorical or metonymical polysemes. In the case of both types of homonyms, a significant N400 priming effect was found for both dominant and subordinate meanings, although subordinate meanings elicited a relatively small effect. These findings were taken to indicate that every meaning is activated in an exhaustive manner for homonyms, but the extent of activation varies based on its frequency and context, at least in short inter-stimulus intervals (ISIs) (but see MacGregor et al., 2015, for the results using long ISIs).

In ERP studies using unprimed LDT reported a facilitative effect of semantic ambiguity with significant N400 modulations by homonyms (Haro et al., 2017) as well as polysemes (Taler et al., 2013; Haro et al., 2017). Haro et al. (2017) compared N400 amplitude elicited by polysemous, homonymous, and unambiguous words in an LDT study, manipulating the number of meanings (NOM) and the relatedness of meanings (ROM) of each word measured in separate subjective ratings. The behavioral results showed faster latencies for both types of ambiguous words, accompanied by an enhancement in N400 amplitude compared to unambiguous words. Based on these findings, the authors concluded that semantic ambiguity facilitates visual word recognition, regardless of the relatedness of meanings, benefiting from a rich amount of semantic activation (Haro et al., 2017). These results were consistent with the findings of Taler et al. (2013), who demonstrated a facilitative effect of polysemous words in an LDT study. Taken together, a body of evidence has demonstrated the significance of the N400 effect in relation to semantic processing during the recognition of ambiguous words. This holds true regardless of their type, the dominance of meaning, or the experimental paradigm used. Here, we aimed to explore whether attenuated or increased N400 amplitude is elicited by balanced homonyms, and if word frequency modulates this effect.

Another relevant ERP component might be P600, alternatively termed the late positivity, a positivity typically emerges from 500 ms and peaks around 600 ms after the stimulus onset. The P600 component, although traditionally linked to the syntactic reanalysis, has also been associated with the reanalysis and integration of semantic information (for a recent review, see Leckey and Federmeier, 2020), in studies using words (Meyer and Federmeier, 2007; MacGregor et al., 2015) as well as sentences (Van Petten and Kutas, 1987; Kim and Osterhout, 2005). With respect to semantic ambiguity, studies using homonyms in sentential context found an equivalent enhancement of P600 amplitude for dominant and subordinate meanings, compared to unrelated trials (Van Petten and Kutas, 1987; Kotchoubey and El-Khoury, 2014). Two studies (MacGregor et al., 2015; Meade and Coch, 2017) on homonyms examined the P600 effect in priming studies using word stimuli, where both dominant and subordinate meanings modulated P600 amplitude, with subordinate pairs eliciting stronger P600 priming effect as compared to dominant ones. However, it remains to be investigated whether cooperative or competitive dynamics of semantic processing can elicit a discrete P600 effect in the late time windows, controlling for meaning frequencies of homonyms. Here, we examined whether the P600 effect would reflect the semantic ambiguity effect at the single-word level, elicited by balanced homonyms, as compared to unambiguous words. Furthermore, the P600 effect has also been suggested to reflect more general cognitive processes such as decision-making and categorization that involve strategic control or task demand (Friederici et al., 1996; Hahne and Friederici, 1999; Royle et al., 2013; Fromont et al., 2020). If the inhibitory effect for homonyms stems from task-specific processing demand, as argued by decision-making accounts (Pexman et al., 2004; Hino et al., 2006), the ambiguity disadvantage would be reflected in the P600 modulation rather than in the N400, which is known to reflect lexical-semantic processes. Thus, we aimed to investigate whether inhibitory effects for homonyms are elicited with an enhanced P600 amplitude, indicating the involvement of decision-making processes during the later stage of processing homonyms.

The present study delves into the temporal unfolding of lexical-semantic processes, using Korean homonymy. Employing the robust methodology of event-related potentials (ERPs) during electroencephalogram (EEG) recording, we scrutinize the temporal dynamics of the lexical and semantic processing during visual word recognition with two pivotal factors: word frequency and lexical ambiguity. To examine whether multiple meanings have an inhibitory or facilitative effect on word recognition, the present study selected balanced homonyms with multiple unrelated meanings. Here, we carried out subjective ratings to measure the number of meanings (NOM), the relatedness of meanings (ROM), and the subjective frequency of each meaning (i.e., meaning balance). Haro and Ferré (2018), who compared two different measures of NOM, found the opposite pattern of results: inhibitory effect of semantic ambiguity for objective measure (i.e., number of dictionary entries) and facilitative effect for subjective measure. To manipulate the semantic ambiguity condition, we considered both subjective and objective measures for NOM, as suggested by Haro and Ferré (2018), while excluding the potential confounding effect of polysemes by controlling the ROM and the subjective balance of homonyms.

To explore the time course of semantic ambiguity, we focused on the two ERP components: the N400 and the P600, which are known to reflect lexical-semantic and post-lexical reanalysis processes, respectively. We hypothesized that, as suggested in previous behavioral findings (Pexman et al., 2004; Kim et al., 2021), homonyms would show divergent effects depending on their word frequency: ambiguity advantage for low-frequency words vs. ambiguity disadvantage for high-frequency words. Further, we hypothesized that, if the facilitative effect of multiple meanings occurs during the stage of lexical access reflected in the word frequency effect, low-frequency unambiguous words would show enhanced N400 amplitude compared to low-frequency homonyms along with behavioral processing advantage reflected in faster latencies and lower error rates. Finally, regarding the inhibitory semantic ambiguity effect for high-frequency words, we investigated whether it occur during the stage of lexical-semantic access in the N400 time window or later during the post-lexical stage in the P600 time window. From the perspective of the accounts that assumed competitive semantic access due to the independent representations of multiple unrelated meanings of homonyms, the ambiguity disadvantage for high-frequency words would modulate the N400 effect. In contrast, according to the decision-making account, these inhibitory effects might occur during the later post-lexical stages only after the lexical access has finished since they were assumed to derive from task-specific processing demand during decision-making rather than the lexical-semantic processes.

## Materials and methods

### Participants

Thirty healthy native speakers of Korean (10 female; 19–30 years old, mean = 23.53 ± 2.9, M ± SD) with normal or corrected-to-normal vision participated in the experiment. All were right-handed, and none reported any neurological or psychiatric impairment history. Participants were informed and provided written consent prior to the experiment and were compensated for their participation. Data from two participants were excluded from the analyses due to excessive noise in the EEG recordings (epoch rejection rate above 50%). Among the remaining 28 participants (19–30 years old, 23.57 ± 2.75), 10 were female. The study was reviewed and approved by the ethics committee of the Korea University Institutional Review Board.

### Materials

Seventy-six target words were selected from the Korean Sejong corpus with a size of 15 million words (Kang and Kim, 2009), which consisted of 36 words with multiple unrelated meanings (i.e., homonyms) and 36 words with single meanings (i.e., control words). The lexical frequency was also manipulated in that each ambiguity condition had two frequency levels, high and low. High frequency words had occurrences above 800, while those with low frequency had occurrences below 10 and above 1. All stimuli set including target words and pseudowords had a length of 3 syllables. Experimental conditions and descriptive statistics of lexical-semantic variables are presented in the Table 1.

**Table 1:** Examples and descriptive statistics of experimental stimuli set.

**Word frequency**	**Lexical ambiguity**	**Example**	**Translation**	**Word Len**	**Log WFPM**	**Obj NOM**	**Subj NOM**	**Subj Bal**
High	Control	사람들 [s^h^aramdɯl]	People	3	3.16 (0.22)	1 (0)	-	-
	Homonym	우리를 [urirɯl]	(1) Us (2) Cage	3	3.18 (0.28)	2.47 (0.84)	2 (0.22)	2.14 (0.45)
Low	Control	은화를 [ɯnhwarɯl]	Silver coin	3	0.7 (0.15)	1 (0)	-	-
	Homonym	전시와 [tsʌnɕiwa]	(1) Exhibition (2) Wartime	3	0.78 (0.2)	2.21 (0.54)	1.97 (0.4)	2.28 (0.19)
Pseudoword		자맴과 [tsamεmgwa]	-	3	-	-	-	-

Standard deviation is presented in the parentheses. Word Len, Word length in syllable; Log Freq, log-transformed word frequency per million; NOM, number of objective meanings; Subj NOM, number of subjective meanings; Subj Bal, subjective balance.

For the selection of homonyms (i.e., words with multiple unrelated meanings), two sets of subjective ratings were carried out to obtain the subjective number of meanings (subj NOM) and their relative frequency of use. One hundred and eighty-six 3-syllable words with two or more objective number of meanings (obj NOM) were selected as candidates from the given corpus. Based on the Korean Standard Dictionary, words that have two or more meanings from a single entry were excluded in order to control for the polysemy effect. Twenty native Korean speakers (10 females; years, 19–29 years, 23.85 ± 3.42, M ± SD) who did not participate in the experiment took part in the subjective ratings. Participants were instructed to produce as many meanings as possible of a presented word in a free form, for instance, synonyms, sentences, and English words. Thereafter, they were asked to decide how frequently a meaning they produced is used in daily life, on a 7-point Likert scale, ranging from 1 (“I have never used this meaning”) to 7 (“I use this meaning very frequently”).

The balance of meanings was calculated by subtracting the subjective frequency of use of the first meaning (i.e., the most frequently used meaning) and that of the last meaning (i.e., the least frequently used meaning) based on the second rating score. The candidates that showed a mean subj NOM below 1.5 and/or a mean balance above 3 in the subjective ratings were excluded. As a result, 38 words with multiple unrelated meanings, including 19 high-frequency words and 19 low-frequency words were selected for homonym condition. Thirty-eight words with single meanings were also selected from the given corpus for the control condition while excluding polysemy based on the Korean Standard Dictionary as in the homonym selection process.

A list of pseudowords was generated for the foils in the lexical decision task (LDT), by concatenating 2 syllables randomly sampled from real stems and 1 syllable from real suffixes A total of 76 pronounceable pseudowords without any meaning were selected from the list. These pseudowords were not found in the Standard Korean Dictionary and adhered to the phonological constraints of Korean.

### Procedure

Participants were seated in a comfortable chair in an electrically shielded and sound-attenuated room, with a screen-to-naison distance of 65 cm. After signing a written consent and being informed of the experiment, the EEG cap was fitted, embedded with 32 Ag/AgCl electrodes connected to the scalp via ionic gel. In a lexical decision task (LDT), participants were instructed to decide whether the stimulus presented at the center of the screen (27-inch monitor, LG 27MK400H with a refresh rate of 60 Hz) was a word or not. The experiment began with 20 practice trials, during which feedback was provided, including the correctness of each trial (labeled as “Correct” or “Incorrect”) and the overall accuracy rate (%) after every trial. Participants who scored above 80% accuracy in the practice trials were instructed to proceed to the experimental trials, which consisted of 152 trials, with a 1-min break halfway through. Experimental trials were conducted as follows. First, a fixation point (^***^) was presented on the center of the screen for 300 ms, followed by a blank screen for 1,500 ms. Then, a target stimulus was presented for 1,500 ms on a black background in a white font (Courier New, 40 point), which was arranged in a fully random order for each participant. Finally, an inter-trial interval (1,500 ms) featured an eye-blink sign (>__ <), during which participants were allowed to blink their eyes. Eye and body movements were discouraged during the rest of the trial to minimize EEG noise. The stimulus duration and presentation were controlled using E-prime 2.0 Professional Software (Psychology SoftwareTools, Inc., Pittsburgh, United States of America).

### EEG acquisition and preprocessing

EEG was recorded from 32 Ag/AgCl electrodes connected to an elastic cap (actiCAP, Brain Products GmbH, Gilching, Germany), following the international 10–20 convention of 32-channel cap (for a detailed location for each electrode, see Figure 4). The eye movement was detected and monitored with a vertical electrooculogram (VEOG) located below the right eye. Signals were referenced online against a dedicated reference electrode mounted on between the Fz and Cz channels, were amplified using a BrainAmp DC amplifier system (Brain Product GmbH, Gilching, Germany) and were digitized with a sampling rate of 1,000 Hz. All impedances were kept below 10 kΩ during the recording session.

The EEG data was preprocessed using a custom-built MATLAB (The MathWorks Inc, 2023) script with EEGLAB toolbox (Version 2023.1; Delorme and Makeig, 2004). EEG signals were first down-sampled to 500 Hz, and 60 Hz line noises were filtered out using CleanLine EEGLAB plugin (Version 2.0). Thereafter, the EEG data was band-pass filtered to 0.1–100 Hz using FIR filter. Bad channels that showed poor correlation with adjacent channels (correlation threshold: 0.7) or noisy signals (noisy line threshold: 2.5) were removed with clean_rawdata EEGLAB plugin (Version 2.8) and were interpolated. EEG signals were then re-referenced offline to the linked mastoids. To detect artifacts including eye and body movements, independent component analysis (ICA) was performed, followed by IC classification using ICLabel EEGLAB plugin (Version 1.4; Pion-Tonachini et al., 2019). ICs that showed probabilities for brain signal below 1% and either probability for eye or muscle movements above 80% were removed, resulting in a mean rejection rate of 3.63%.

IC-rejected EEG signals were band-pass filtered again to 1–30 Hz using FIR filter for ERP analyses. The continuous EEG data was segmented into 152 epochs with a length of 1,100 ms (−100–1,000 ms post-stimulus) time-locked to the target onset and baseline-corrected using the pre-stimulus interval of 100 ms. The residual artifact was removed by rejecting epochs where any channel showed amplitude exceeding ±100 μV, resulting in a mean epoch rejection rate of 15.62%.

### Statistical analyses

The response time (RT) and error data were analyzed with linear mixed effects model (Baayen et al., 2008) using lme4 package (Version 1.1.35; Bates et al., 2015) in R software (Version 4.3.1; R Core Team, 2023). Only correct responses were included in the RT analysis, and raw RTs were log-transformed to address the skew of RT distribution. Error data was modeled with a binomial distribution using glmer function. For both analyses, two fixed factors Frequency (2: High vs. Low) and Ambiguity (2: Control vs. Homonym) with random intercepts of both participant and item were included to take individual differences of participants and variance across items into account. A *post-hoc* comparison was carried out using estimated marginal means with Bonferroni correction for multiple comparisons, in which adjusted *p*-values are reported.

**Figure 1:**
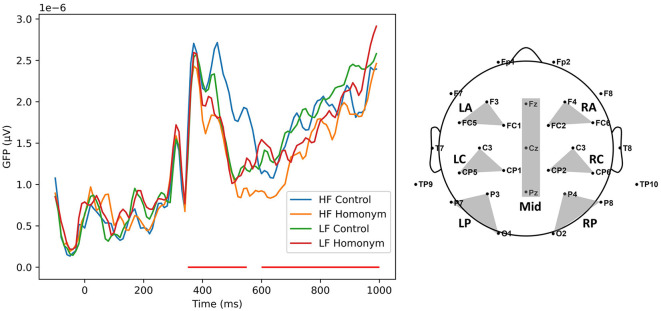
**(Left)** A plot of Global field power (GFP) of the four conditions. The red lines denote the time windows of interest (N400: 350–550 ms; P600: 600–1,000 ms). **(Right)** The montage for 32 channel EEG recording. The shaded areas represent seven regions of interest (ROIs): LA (left anterior), RA (right anterior), LC (left central), RC (right central), LP (left parietal), RP (right parietal), and Mid (midline). HF, high-frequency; LF, low-frequency.

To determine time windows of interest (TOIs) of the ERP data, we first plotted a plot of the Global Field Power (GFP) for each condition (see Figure 1). The GFP measure offers an unbiased insight into component latencies, as it takes into account topographical similarities across all recordings from each electrode simultaneously (Skrandies, 1990). Three time windows were chosen for each component based on the GFP measure and the prior research on visual recognition of Korean words (Kang et al., 2016; Kim et al., 2022): 350–550 ms, and 600–1,000 ms for N400 and P600, respectively. Thereafter, epochs of each participant were averaged over five experimental conditions and regions of interest (ROIs; see Figure 1 for detailed electrode locations) along one midline (Fz, Cz, and Oz) and six lateral (left anterior: F3, FC1, FC5, right anterior: F4, FC2, FC6, left central: C3, CP1, CP5, right central: C4, CP2, CP6, left posterior: P3, P7, O1, right posterior: P4, P8, O2) regions. As in the RT analysis, only correct responses were included in a series of ERP analyses. The grand-averaged ERP data were submitted to separate repeated measures ANOVAs for lateral and midline ROIs. For lateral ROIs, analyses were run with two experimental factors Frequency (Low vs. High) and Ambiguity (2: Control vs. Homonym), and two topographical factors Hemisphere (2: Left vs. Right) and Column (3: Anterior vs. Central vs. Posterior), while factors Frequency, Ambiguity, and Column were involved for midline analysis. Here, a Greenhouse-Geisser correction for a lack of sphericity was applied when applicable (i.e., *p* < 0.05 in Mauchly's sphericity test), and adjusted degrees of freedom, *p*-values, and effect sizes measured with generalized eta squared ($\eta_{G}^{2}$) are reported. In case a significant main effect of an experimental factor or an interaction with an experimental factor was found, a region-wise pairwise *t*-test with Bonferroni correction for multiple comparisons was performed. For midline ROI, the same *post-hoc* pairwise *t*-test was run on each electrode (Fz, Cz, and Pz).

In addition, to obtain insight into the spatial and temporal distribution in which differences emerged, we carried out a non-parametric cluster-based permutation test (Maris and Oostenveld, 2007), which addresses the multiple comparison problem without reducing the statistical power. Each pairwise comparison (HF vs. LF, Control vs. Homonym, HF: Control vs. Homonym, and LF: Control vs. Homonym) was submitted to a two-tailed cluster-level paired *t*-test with 2000 randomizations, as implemented in MNE-Python software (Gramfort et al., 2013). Clusters were formed based on spatio-temporal adjacency of electrodes, in which neighboring electrodes were defined using the triangulation algorithm, and only those with permutation *p*-value below the cluster alpha level (*p* < 0.05) were selected. Finally, the clusters with *p*-values below 0.025 from the two-tailed *t*-tests were obtained. R software (Version 4.3.1; R Core Team, 2023) and MNE-python (Gramfort et al., 2013) were used for statistical analyses and visualization.

## Results

### Behavioral data

**Figure 2:**
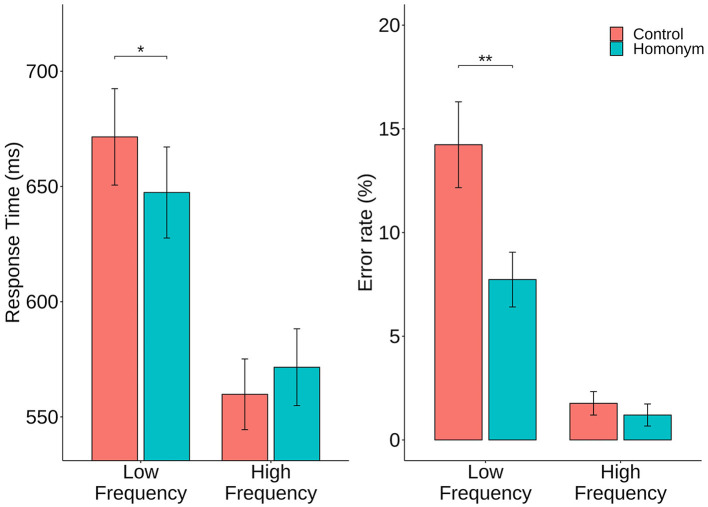
Mean response times (RTs) in ms and % error rates for the four conditions. Error bars denote standard error and asterisks indicate statistical significance in *post-hoc t*-tests (^*^p < 0.05, ^**^p < 0.01).

The descriptive statistics are shown in Table 2. The outlier trials with RTs exceeding ±3 standard deviations from the mean or those with error rates above 20% were excluded from further analyses, resulting in a total of 7.32% exclusion rate. Raw RTs were used for visualization purposes (see Figure 2) and log-transformed RTs were calculated and used throughout the analyses to address distribution skew. Overall, participants made lexical decisions with an average RT of 649.1 ms, a log-transformed RT of 2.8 ms, and an error rate of 7.39%.

**Table 2:** Descriptive statistics of behavioral data.

	**Word frequency**					
	**Low**			**High**		
**Lexical ambiguity**	***Mean RT***	***Mean Log RT***	***% Error***	***RT***	***Log RT***	***% Error***
Control	677.4 (21)	2.82 (0.01)	15.71 (2.48)	563.14 (15.15)	2.74 (0.01)	1.7 (0.55)
Homonym	652.05 (19.6)	2.8 (0.01)	8.96 (1.77)	575.59 (16.56)	2.75 (0.01)	1.35 (0.53)

SEM (standard error of the mean) is presented in the parentheses. RT, raw response time (ms); Log RT, log-transformed response time (ms); % Error, error rate (%).

Analysis of RT data demonstrated a significant main effect of Frequency [*b* = 0.03, *SE* = 0.003, *t* = 9.75, *p* < 0.001], with high-frequency (HF) words showing faster response compared to low-frequency words [LF vs. HF: 2.81 vs. 2.74 ms, *b* = 0.07, *SE* = 0.01, *t* = 9.53, *p* < 0.001]. Although the main effect of Ambiguity failed to reach significance, a critical interaction of Frequency and Ambiguity showed trend-toward-significance [*b* = 0.007, *SE* = 0.003, *t* = 1.93, *p* = 0.058]. *Post-hoc* comparisons on this interaction showed that participants were marginally faster to decide the lexicality of homonyms when targets were LF words [LF Control vs. LF Homonym: 2.82 vs. 2.8 ms, *b* = −0.019, *SE* = 0.01, *t* = 1.9, *p* = 0.061], while no significant difference was found between control words and homonyms in HF condition [HF Control vs. HF Homonym: 2.74 vs. 2.75 ms, *b* = −0.008, *SE* = 0.01, *t* = −0.75, *p* = 0.45].

The error data also yielded a significant main effect for Frequency [*b* = 1.16, *SE* = 0.17, *z* = 6.68, *p* < 0.001]. Specifically, low-frequency words resulted in a higher error rate compared to those with high frequency [LF vs. HF: 12.33 vs. 1.52%, *b* = 2.33, *SE* = 0.35, *z* = 6.68, *p* < 0.001]. In addition, although an interaction between Frequency and Ambiguity was not significant, *post-hoc* comparisons were carried out for an exploratory purpose. Here, as in RT analysis, participants yielded more errors in control words compared to homonyms, only in the LF condition [LF Control vs. LF Homonym: 15.71 vs. 8.96%, *b* = 0.72, *SE* = 0.35, *z* = 2.05, *p* = 0.04], but not in the HF condition [HF Control vs. HF Homonym: 1.7 vs. 1.35%, *b* = 0.25, *SE* = 0.59, *z* = 0.42, *p* = 0.68].

### Electrophysiological data

**Figure 3:**
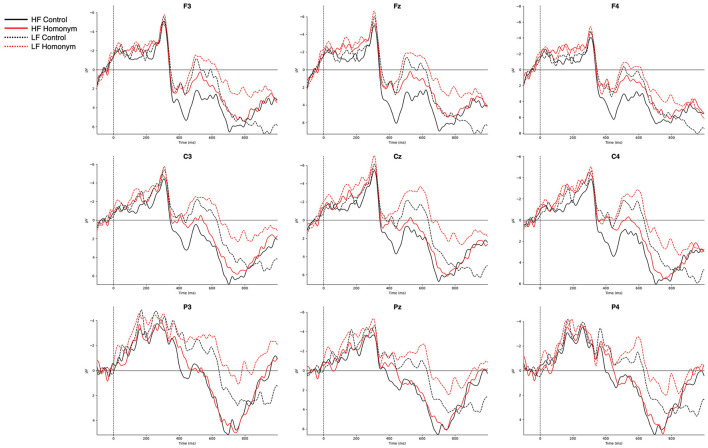
Grand-averaged ERPs for the four conditions from −100 to 1,000 ms after the target onset. Nine representative electrodes from frontal (F3, Fz, F4), central (C3, Cz, C4), and parietal (P3, Pz, P4) regions are presented. Negative amplitudes are plotted upwards. HF, high-frequency; LF, low-frequency.

**Figure 4:**
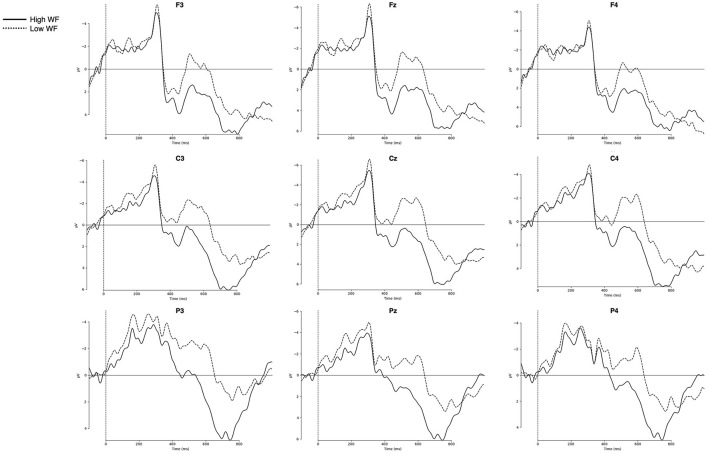
Grand-averaged ERPs for the high-frequency and low-frequency words. Nine representative electrodes from frontal (F3, Fz, F4), central (C3, Cz, C4), and parietal (P3, Pz, P4) regions are presented. Negative amplitudes are plotted upwards. WF, word frequency.

**Figure 5:**
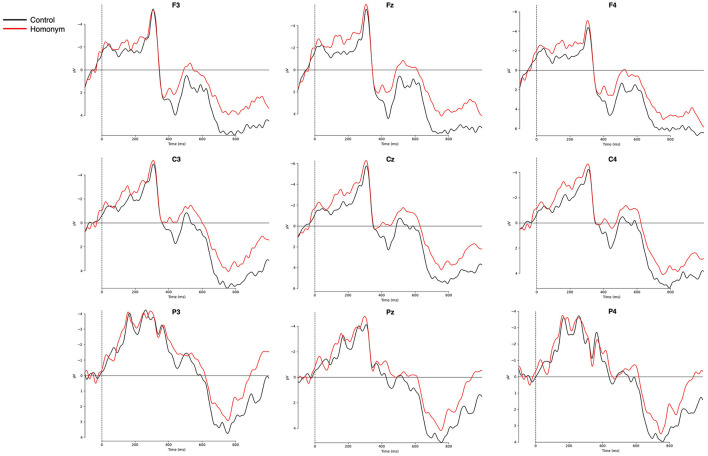
Grand-averaged ERPs for the homonyms and control (i.e., unambiguous) words. Nine representative electrodes from frontal (F3, Fz, F4), central (C3, Cz, C4), and parietal (P3, Pz, P4) regions are presented. Negative amplitudes are plotted upwards.

Figure 3 represents the grand-averaged ERPs evoked by each condition on representative channels. Comparisons of ERP curves for the effects of word frequency and ambiguity are shown in Figures 4, 5, respectively. As shown in the Figures 3–5, a negative peak and a positive peak are apparent in 350–550 ms (N400) and 600–1,000 ms (P600), respectively.

In the two pre-defined time windows, we conducted repeated measures ANOVAs with the experimental factors Frequency (HF vs. LF) and Ambiguity (Control vs. Homonym), as well as the topographical factors Hemisphere (Left vs. Right) and Column (Anterior vs. Central vs. Posterior) for lateral regions of interest (ROIs). Additionally, for the midline ROI, we employed the factors Frequency, Ambiguity, and Column. A *post-hoc* pairwise *t*-test was performed in case any significant effect was found. If an experimental factor showed interaction with topographical factor(s), a region-wise *t*-test was conducted on each region for lateral analyses, and on each channel for midline analysis. In any *post-hoc t*-test, *p*-values were adjusted with Bonferroni correction for multiple comparisons. For a repeated measures ANOVA, adjusted degrees of freedom and *p-*values with Greenhouse-Geisser correction are reported when applicable. Here, only effects associated with the experimental factor(s) are reported in repeated measures ANOVA results.

#### N400 (350–550 ms)

**Figure 6:**
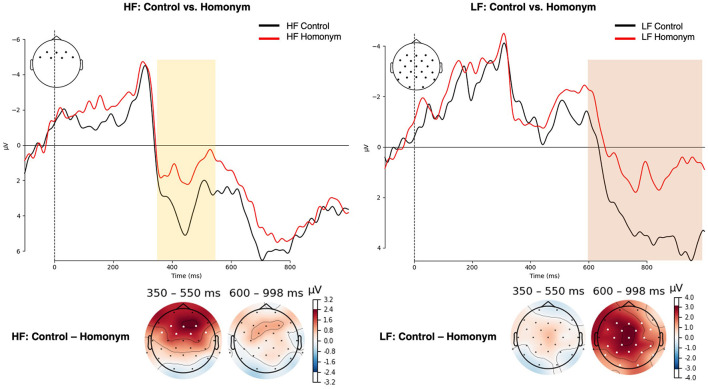
**(Top)** ERP waveforms averaged over electrodes that showed statistical significance in region-wise *post-hoc t*-tests for the semantic ambiguity effect within each word frequency condition. **(Bottom)** Topographical distributions representing the semantic ambiguity effect within each word frequency condition. Negative amplitudes are plotted upwards, and time windows (HF: 350–550 ms; LF = 600–1,000 ms) and electrodes presenting a significant difference in *post-hoc t*-tests are highlighted. HF, high-frequency; LF, low-frequency.

In the N400 time window (350–550 ms), word frequency yielded a significant main effect was observed for both lateral and midline ROIs [lateral: *F*_(1, 27)_ = 8.91, *p* = 0.006, η^2^_*G*_ = 0.038; midline: *F*_(1, 27)_ = 12.43, *p* = 0.002, η^2^_*G*_ = 0.055], with low-frequency words eliciting more negative-going amplitude (see Figure 4) [LF vs. HF, lateral: *t*_(55)_ = −3.1, *p* = 0.003; midline: *t*_(55)_ = −3.79, *p* < 0.001]. Although there was no significant main effect of Ambiguity, Ambiguity × Column interaction was significant for lateral ROIs [*F*_(1.24, 33.42)_ = 14.21, *p* < 0.001, η^2^_*G*_ = 0.003] and yielded a trend-toward-significance for midline ROIs [*F*_(1.27, 34.22)_ = 3.33, *p* = 0.068, η^2^_*G*_ = 0.002]. The *post-hoc* region-wise *t*-tests showed that reduced N400 amplitude for homonyms than control words in fronto-central regions [Fz: *t*_(55)_ = 2.13, *p* = 0.038; Cz: *t*_(55)_ = 2.01, *p* = 0.049]. More importantly, a critical Frequency × Ambiguity × Column interaction was significant for midline electrodes [*F*_(2, 54)_ = 6.29, *p* = 0.004, η^2^_*G*_ = 0.002] and marginally significant for lateral electrodes [*F*_(1.24, 34.44)_ = 3.68, *p* = 0.054, η^2^_*G*_ = 0.001]. The *post-hoc* region-wise *t*-tests showed that only in the high-frequency condition, homonyms yielded larger negativity than control words in the N400 latency range, specifically focused on the frontal regions [LA: *t*_(27)_ = 2.16, *p* = 0.04; RA: *t*_(27)_ = 2.22, *p* = 0.035; Fz: *t*_(27)_ = 2.44, *p* = 0.022; see Figure 6].

#### P600 (600–1,000 ms)

In the P600 time window, the main effect of frequency was observed for both lateral and midline ROIs [lateral: *F*_(1, 27)_ = 5.71, *p* = 0.024, η^2^_*G*_ = 0.015; midline: *F*_(1, 27)_ = 4.38, *p* = 0.046, η^2^_*G*_ = 0.011], in which low-frequency words showed attenuated amplitude compared to high-frequency words (Figure 4) [lateral: *t*_(55)_ = −3.1, *p* = 0.003; midline: *t*(55) = 3.79; *p* < 0.001]. In addition, a main effect of Ambiguity was found for midline electrodes [*F*_(1, 27)_ = 9.28, *p* = 0.005, η^2^_*G*_ = 0.02], and for lateral electrodes, both the main effect of Ambiguity [*F*_(1, 27)_ = 7.57, *p* = 0.01, η^2^_*G*_ = 0.014] and the Ambiguity × Column interaction [*F*_(1.43, 38.7)_ = 5.27, *p* = 0.017, η^2^_*G*_ = 0.001] were observed. As shown in Figure 5, control words elicited more positive-going amplitude compared to homonyms [lateral: *t*_(55)_ = 2.64, *p* = 0.011; midline: *t*_(55)_ = 2.9, *p* = 0.005]. Furthermore, a critical Frequency × Ambiguity interaction was found for lateral ROIs [*F*_(1, 27)_ = 4.48, *p* = 0.044, η^2^_*G*_ = 0.001], in which only low-frequency showed a significant difference between control words and homonyms (Figure 6), with control words showing a larger positivity compared to homonyms [*t*_(27)_ = 3.49; *p* = 0.002]. This P600 effect, reflecting the ambiguity effect in low-frequency words, was more widely distributed, while the N400 effect, reflecting the ambiguity effect in high-frequency words, was focused on frontal regions.

#### Cluster-based permutation tests

**Figure 7:**
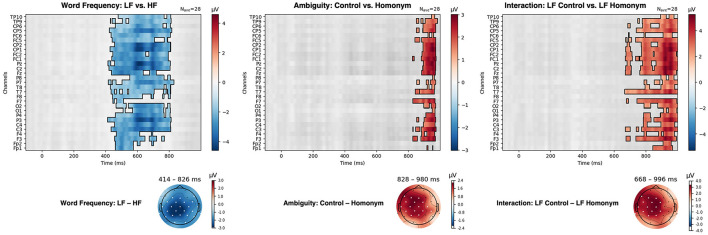
Results of the cluster-based permutation test for low-frequency vs. high-frequency words (LF vs. HF), unambiguous vs. homonymous words (Control vs. Homonym) and low-frequency unambiguous words vs. low-frequency homonyms (LF Control vs. LF Homonym).

A non-parametric cluster-based permutation test was carried out on each pairwise comparison including (1) LF vs. HF, (2) Control vs. Homonym, (3) HF Control vs. HF Homonym, and (4) LF Control vs. LF Homonym. The results of the test are presented in Figure 7, demonstrating statistical significance on every channel except for EOG and mastoid channels (29 Ag/Agcl electrodes) and at every time point (−100–1,000 ms post-stimulus). Here, the topographies of clusters that yielded *p*-values below the critical alpha value of 0.025 (two-tailed) are also presented.

A cluster-based permutation test showed a Word frequency effect (LF vs. HF) in 414–826 ms after the target onset (cluster *p* = 0.001). Consistent with the ANOVA results in the N400 time window (350–550 ms, see Figure 4), enhanced negativity for LF compared to HF words was observed in the widely distributed regions, spanning anterior, central, and parietal regions. Additionally, a pronounced positivity for control words compared to homonyms was displayed, from 828 ms to 980 ms (cluster *p* = 0.024) as in the ANOVA results in the P600 time window (600–1,000 ms, see Figure 5). Although the Ambiguity effect in HF words did not reach statistical significance, the difference between control and homonym words in LF words showed earlier onset compared to the main effect of Ambiguity, from 668 ms to 996 ms (cluster *p* = 0.004). Here, LF homonyms elicited attenuated negativity compared to LF control words as in the region-wise *t*-test results in the P600 time window shown in Figure 6.

## Discussion

The present study aimed to investigate the role and temporal dynamics of the semantic ambiguity effect during the processing of balanced homonyms in insolation, using both behavioral and EEG measures in a lexical decision task. First, we examined whether homonyms demonstrate facilitative or inhibitory effect compared to unambiguous words, and whether this effect diverges depending on the word frequency. The behavioral results indicated that in low-frequency words, homonyms elicited faster and more accurate responses. However, this facilitative effect was not observed in high-frequency words. Furthermore, we sought to explore the time course of the semantic ambiguity effect and its potential interaction with the word frequency, focusing on the N400 and late positivity component. The electrophysiological results revealed a word frequency effect both in the N400 and P600 components, in which an enhanced amplitude was observed for low-frequency words, suggesting a processing demand for less frequent words. In these time windows, we also found a significant interaction of word frequency and semantic ambiguity, where an enhanced N400 amplitude (i.e., increased negativity) was observed for homonyms in high-frequency words, consistent with previous findings on balanced homonyms (Maciejewski and Klepousniotou, 2020). In contrast, the ambiguity advantage for low-frequency words was reflected in a reduced amplitude for homonyms in the subsequent P600 time window. These P600 modulations by the semantic ambiguity for the low-frequency words was observed both in the predefined time window and in non-parametric cluster-based permutation tests.

One of the core findings of the present study is the differential impact of semantic ambiguity on low-frequency and high-frequency words. Consistent with previous studies that manipulated the word frequency of homonyms (Pexman et al., 2004; Kim et al., 2021), we observed a facilitative effect of semantic ambiguity in low-frequency homonyms, which manifested as faster recognition times and an attenuated P600 amplitude. This facilitative effect aligns with the assumption that ambiguous words can access a richer semantic network, thereby enhancing cognitive processing. In contrast, high-frequency homonyms did not exhibit this advantage, suggesting that the facilitative effects of semantic ambiguity are mitigated or even reversed as word frequency increases. Indeed, we found that in high-frequency words, homonyms elicited an increased negativity in the N400 time window.

The enhanced N400 amplitude for homonyms in high-frequency words, albeit the absence of behavioral evidence indicating the ambiguity disadvantage for high-frequency words, might suggest a heightened semantic processing demand, possibly due to the competition of multiple meanings activated during semantic access, as suggested in the predictions of connectionist models (Rodd et al., 2002, 2004; Armstrong and Plaut, 2016). It is also noteworthy that although Taler et al. (2013) observed an N400 reduction for polysemes while Haro et al. (2017) found an enhanced N400 for both types of ambiguous words (i.e., polysemes and homonyms), two studies interpreted these reversal effects as evidence for the facilitative effect of ambiguous words.

Our N400 findings in high-frequency words are consistent with the findings of Maciejewski and Klepousniotou (2020), who observed increased frontal negativity in the N400 time window (350–500 ms after the prime onset) for balanced homonymous primes, but not for biased ones, in an ERP priming study. These results were interpreted to be evidence against the decision-making account (e.g., Pexman et al., 2004), in that ambiguity disadvantage was observed in the semantic activation process reflected in the N400 component during the presentation of ambiguous prime, rather than during the processing or responding to the target word. Additionally, based on their findings in the frontal negativity elicited by balanced homonyms, they argued for the potential involvement of the inhibitory competition resolution process, as suggested in the functional magnetic resonance imaging (fMRI) studies of ambiguity (Bilenko et al., 2008; Klepousniotou et al., 2014). As illustrated in Figure 6, the frontal distribution of the N400 effect aligns with our findings in high-frequency words. These patterns in the topographical distribution suggest that the multiple meanings of high-frequency homonyms may have undergone a competitive process during semantic access prior to reaching the point of lexical decision, considering the possibilities that the facilitative effects found for low-frequency homonyms might have been canceled out, leading to the null effect for high-frequency words in the behavioral results.

In contrast, we found a facilitative effect of semantic ambiguity for low-frequency words both in the behavioral and ERP results. These findings were consistent with the studies that employed homonyms not only in Indo-European languages such as English (Pexman et al., 2004) and Spanish (Haro and Ferré, 2018), but also in non-Indo-European languages, including Chinese (Lin and Ahrens, 2010), Japanese (Hino et al., 2006), and Korean (Kim et al., 2021; Lim and Choi, 2023). Furthermore, it is noteworthy that this ambiguity advantage effect was observed for balanced homonyms, which have meanings with equivalent individual frequency. This aligns with previous studies that found a facilitative effect of semantic ambiguity in balanced homonyms (Haro and Ferré, 2018; Lim and Choi, 2023).

These findings challenge the assumptions that varying semantic settling and processing times for different types of ambiguous words result in the competitive dynamics of processing unrelated meanings of homonyms and cooperative ones for polysemes (Rodd et al., 2002, 2004; Armstrong and Plaut, 2016). Instead, they provide empirical evidence for the connectionist approach, in which multiple meanings benefit from an increase in global activation or semantic feedback to the orthographic level (e.g., Balota et al., 1991; Borowsky and Masson, 1996). More recently, Haro and Ferré (2018) suggested that the semantic ambiguity advantage observed in the LDT can also be explained in terms of the PDP model (Kawamoto, 1993; Kawamoto et al., 1994). They argued that the blend state where vague information from multiple meanings is activated, might be reached faster by the network than the stable state as a result of semantic settling, which is sufficient to make a decision on the lexicality of a presented stimulus in an LDT. These possibilities were also previously discussed in the commentaries on the PDP model, and later confirmed in a simulation study (Borowsky and Masson, 1996).

Furthermore, a reverse pattern in the P600 component for low-frequency words (i.e., an attenuated P600 amplitude for homonyms compared to unambiguous words) corroborates a facilitative effect of semantic ambiguity for low-frequency words, aligning with our behavioral findings. The previous ERP literature has associated the P600 or late positivity complex (LPC) with syntactic or grammatical processes, in which an enhanced P600 amplitude was thought to indicate costs from post-lexical reanalysis (Friederici, 1995; Münte et al., 1997) or syntactic integration (Hagoort and Brown, 2000; Kaan et al., 2000). However, alternative functional accounts have also been proposed, that semantic aspects of the P600 should also be considered (Kuperberg et al., 2003; Van Herten et al., 2005), where the P600 enhancement was taken to indicate processing demand stemming from semantic anomalies or semantic integration (for recent reviews on the semantic P600, see Van Petten and Luka, 2012; Leckey and Federmeier, 2020). Considering the general association of an increased P600 with processing demand, regardless of whether it stems from syntactic or semantic processing, our behavioral and P600 findings corroborate an ambiguity advantage effect, at least for low-frequency words.

The previous ERP studies on ambiguous words have demonstrated the P600 component modulated by homonyms, mainly in sentential context (e.g., Van Petten and Kutas, 1987; Swaab et al., 2003), but also in word priming (i.e., word-pair) paradigm (MacGregor et al., 2015; Meade and Coch, 2017). An increased P600 for homonyms was observed in the studies that employed sentences (Swaab et al., 2003; Meyer and Federmeier, 2007; MacGregor et al., 2015) and long SOAs in the word-pair priming paradigm (MacGregor et al., 2015). Alongside the N400 enhancement for homonyms, these late positivity findings were taken as evidence for the difficulties elicited during the activation, selection, and relating of the meanings of a homonym.

On the other hand, a recent primed LDT study by Meade and Coch (2017) found a reduced amplitude in the late positivity (500–800 ms) for both dominant and subordinate meanings (i.e., related) compared to unrelated primes followed by homonymous targets, using a short SOA word priming paradigm. These were consistent with our findings (i.e., reduced amplitude for homonyms compared to unambiguous words), particularly considering that both studies examined the semantic ambiguity effect at a word level with minimal context. Meade and Coch (2017) claimed that these results reflect post-lexical reprocessing rather than lexical processing, while MacGregor et al. (2015) who employed the long SOA priming paradigm, argued for a decay of both dominant and subordinate meanings in the late time window, preceded by the competition in the N400 time window.

However, our P600 findings cannot be fully explained by either interpretation, taking into account that the word frequency effect indicating lexical processing was also found in the late time window, and that competition indexed by the inhibitory effect in the N400 time window was observed only for high-frequency homonyms, but not for low-frequency ones. Furthermore, these findings challenge the decision-making account (e.g., Piercey and Joordens, 2000), which argued that the ambiguity disadvantage effect emerges at the difficulties in the post-lexical decision-making processes, limited to more semantically demanding tasks (e.g., semantic relatedness judgment). Alternatively, we demonstrated a facilitative effect of homonyms in the late time window, in particular employing EEG measures with superior temporal resolution. These findings suggest that both facilitative and inhibitory effects of semantic ambiguity might occur at the stage of lexical-semantic processing indexed by the lexical access reflected in the word frequency effect, rather than task-specific decision-making demand.

With respect to the discrepancy in the direction of the semantic ambiguity effect, Pexman et al. (2004) argued that slower latencies for low-frequency words enable ample time to benefit from semantic feedback to orthographic activation. In contrast, the lexical decision on high-frequency words is made prior to this semantic feedback, resulting in the absence of a facilitative effect. This interpretation seems plausible since our behavioral results displayed similar patterns as in Pexman et al. (2004). However, our ERP results, in particular the N400 enhancement observed solely for high-frequency words, suggest that not only the absence of benefit from semantic feedback but also the competition among meanings might occur for high-frequency words. An alternative explanation would be the individual meanings of high-frequency words, which have high meaning frequency in nature compared to low-frequency meanings of low-frequency words. These high-frequency meanings may incur competitive processes, as observed particularly in the case of balanced homonyms (Rayner and Duffy, 1986; Duffy et al., 1988; Kawamoto, 1993). In contrast, lexical information of low-frequency words might not be sufficient for making decision on lexicality in an LDT, thus lending support from either global activation gains or semantic feedback, elicited by individual meanings with low frequencies.

In the present study, we observed the word frequency effect in the N400 time window, which appears later than the early effects reported in many other studies, which have indicated that the word frequency effect takes place much earlier, even before 200 ms (Sereno and Rayner, 2003; Hauk et al., 2006). A possible explanation for the discrepancy in the timing of word frequency effects might be the differences in linguistic structure between languages. For example, languages with more complex morphological structures, such as Korean, an agglutinative, highly-inflective language, might show different processing timelines. Such cross-linguistic variations can affect how quickly word frequency effects emerge in ERP studies, which is supported by findings from Barber and Kutas (2007), who discuss how language-specific factors can influence ERP patterns, and Hauk et al. (2009), who showed variability in ERP responses based on linguistic context. Additionally, it is important to note that some studies have not shown significant differences in the early time window (N1) for word frequency effects. For instance, Barber and Kutas (2007) and Hauk et al. (2009) have observed that word frequency effects can manifest in later ERP components, such as the N400, indicating that these effects may vary depending on the specific lexical and semantic processing demands of the task. Given these considerations, the observed discrepancy in the timing of the word frequency effect highlights the importance of task and stimulus characteristics in ERP studies. Future research should aim to systematically vary these factors to better understand their influence on the timing of lexical processing effects.

The present study is not without its limitations. We observed that the mean subjective NOM for low-frequency homonyms was slightly below 2 (1.97) (see Table 1), indicating that some participants rated these homonyms as having only one meaning. This could be problematic if the participants who performed the lexical decision task (LDT) also perceived some homonyms as having a single meaning as this would diminish the intended semantic ambiguity of these stimuli. It is important to note that all homonyms used in this study had an objective NOM above 2 according to the Standard Korean Dictionary and the corpus. However, since we did not collect subjective NOM ratings from the participants who performed the LDT, it is possible that some homonyms did not effectively induce semantic ambiguity for all participants. Future studies should consider including a subjective NOM rating task for the same participants who perform the LDT to ensure that the stimuli are perceived as ambiguous and to better control for individual differences in processing semantic ambiguity.

## Conclusion

In summary, the present study investigated the semantic ambiguity effect and the time course of lexical and semantic processing, employing both behavioral and electrophysiological measures. While low-frequency homonyms yielded a facilitative effect both in behavioral and P600 results, high-frequency homonyms displayed an inhibitory effect in the N400 time window. Thus, we concluded that the activation of semantic information occurs simultaneously with the lexical processing, rather than during the post-lexical or decision-making processes and that the differential semantic ambiguity effect depending on the word frequency may result from the discrete individual frequency of meanings. Our findings contribute to a more granular understanding of semantic ambiguity, challenging simplistic notions of how semantic ambiguity operates across different lexical frequency and offering new perspectives for future research. Furthermore, the focus on Korean homonymy, with its unique properties, suggests that the temporal dynamics underlying the lexical-semantic processing in Korean may have both commonalities and specificities, prompting further cross-linguistic studies.

## Data availability statement

The raw data supporting the conclusions of this article will be made available by the authors, without undue reservation.

## Ethics statement

The studies involving humans were approved by Korea University Institutional Review Board. The studies were conducted in accordance with the local legislation and institutional requirements. The participants provided their written informed consent to participate in this study.

## Author contributions

JK: Data curation, Investigation, Visualization, Writing – original draft. SK: Methodology, Writing – review & editing. KN: Conceptualization, Supervision, Writing – review & editing.
